# Supplementary figures and images for: A lifecourse mendelian randomization study highlights the long-term influence of childhood body size on later life heart structure
Source: PLoS Biol. 2022 Jun 9;20(6):e3001656. doi: 10.1371/journal.pbio.3001656 (PMC9182693; doi:10.1371/journal.pbio.3001656)

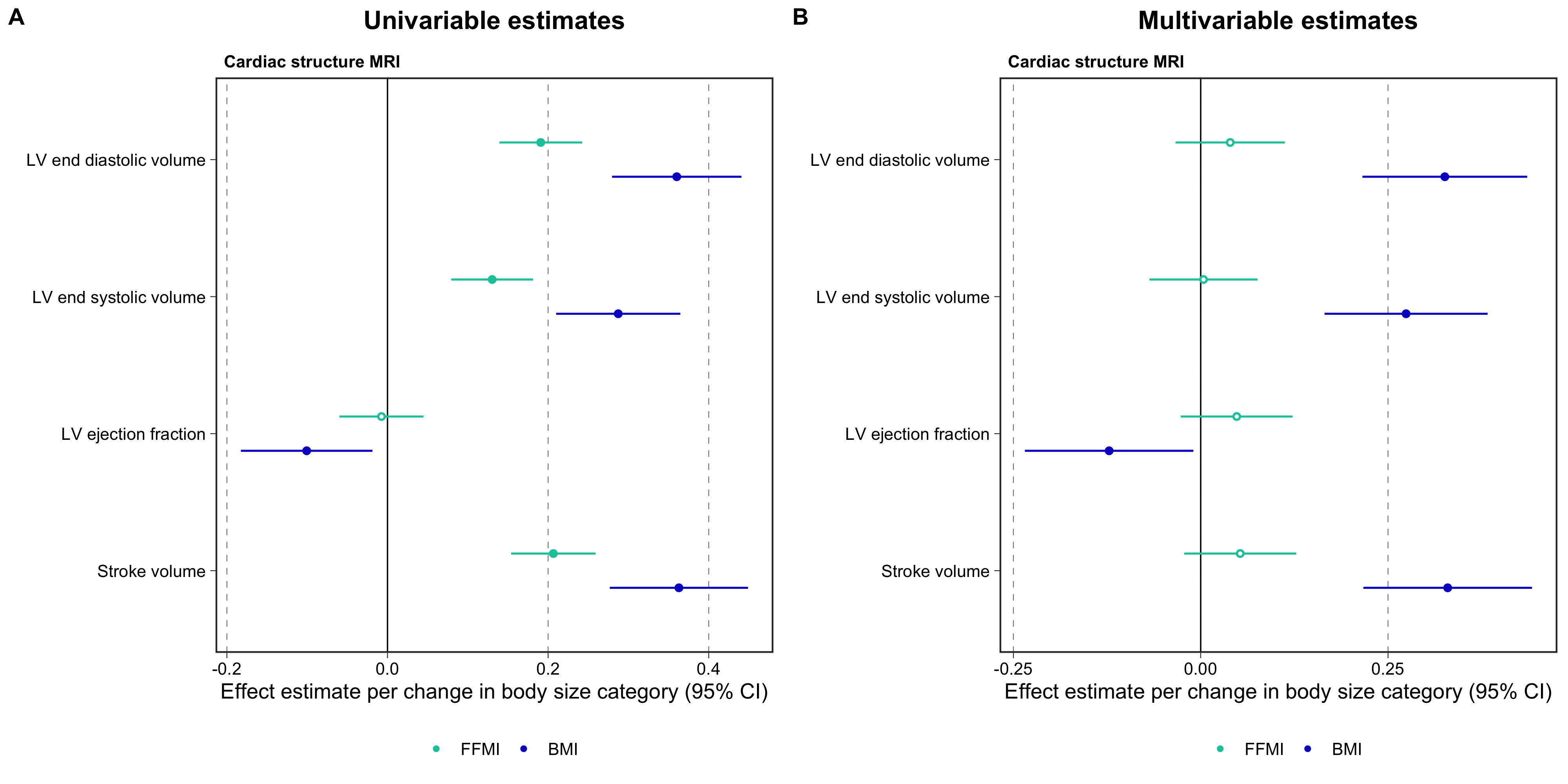

Supplement: S1 Fig — Forest plots illustrating (A) univariable and (B) multivariable MR effect estimates of childhood body size BMI and FFMI on measures of cardiac structure and function. The data underlying this figure can be found in S8 and S9 Tables. BMI, body mass index; FFMI, fat-free mass index; LV, left ventricular; MR, mendelian randomization; MRI, magnetic resonance imaging. (PNG) [file pbio.3001656.s001.png]

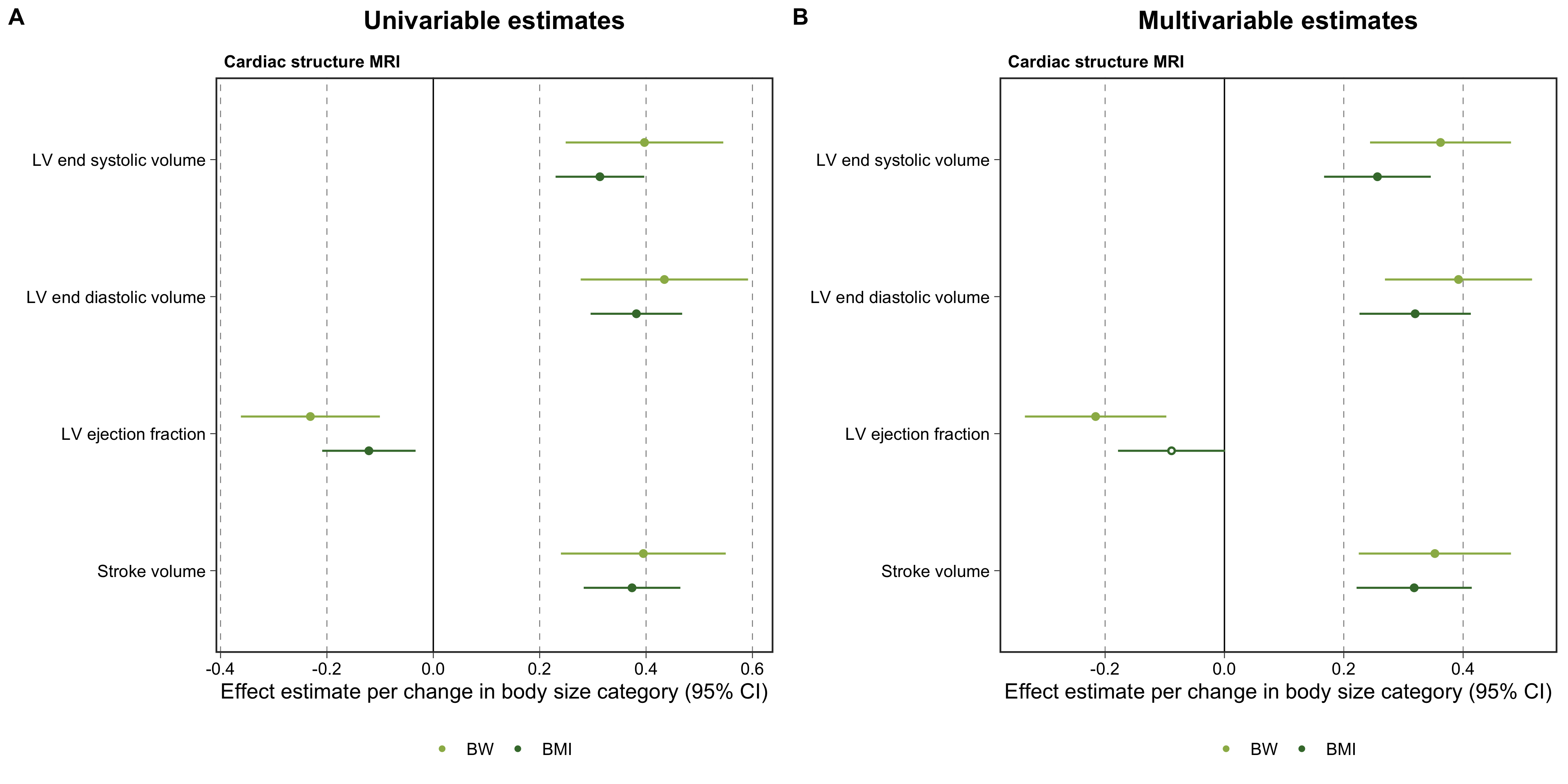

Supplement: S2 Fig — Forest plots illustrating (A) univariable and (B) multivariable MR effect estimates of childhood body size BMI and BW on measures of cardiac structure and function. The data underlying this figure can be found in S10 and S11 Tables. BMI, body mass index; BW, birth weight; LV, left ventricular; MR, mendelian randomization; MRI, magnetic resonance imaging. (PNG) [file pbio.3001656.s002.png]

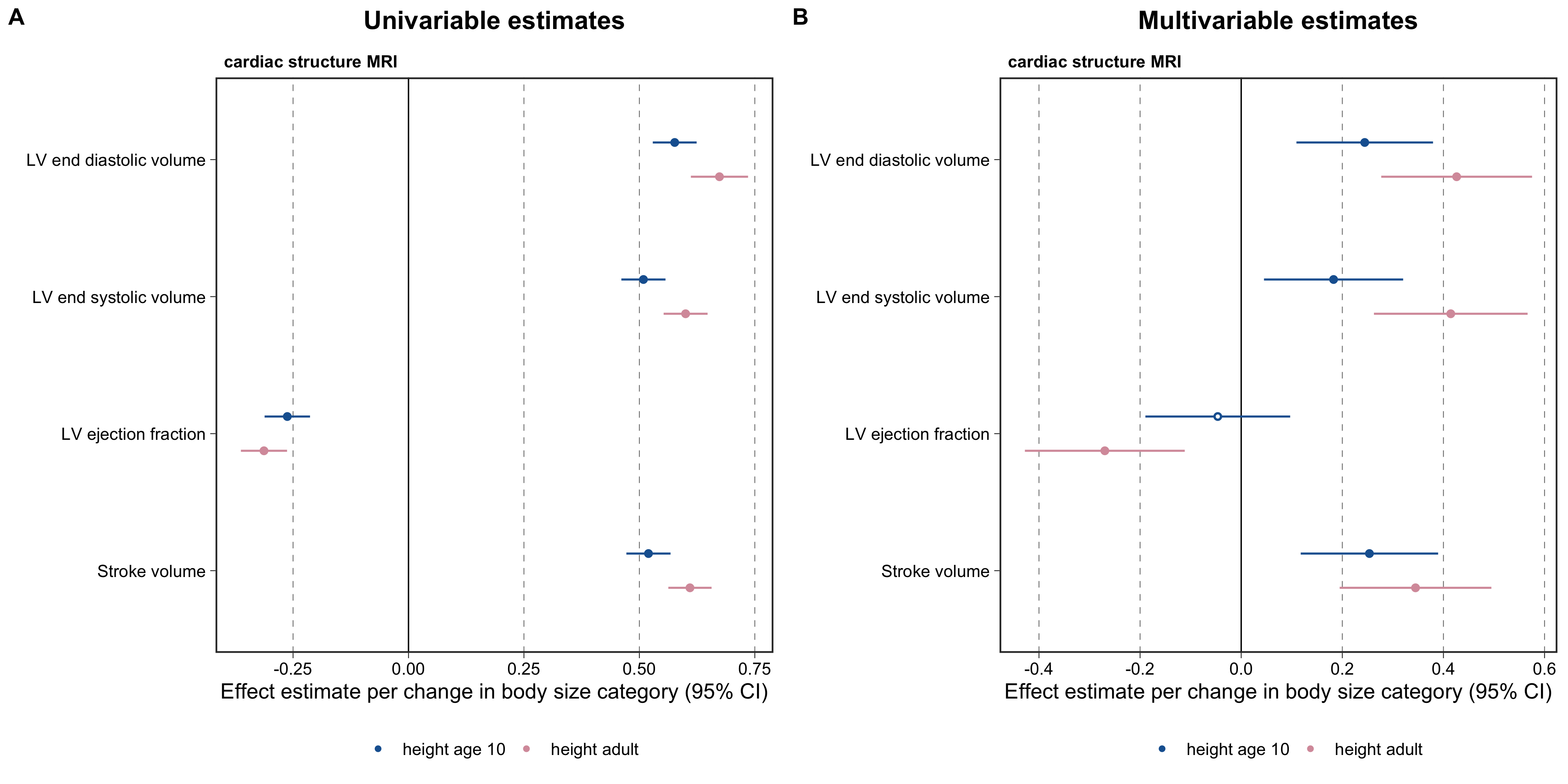

Supplement: S3 Fig — Forest plots illustrating (A) univariable and (B) multivariable MR effect estimates of childhood and adult height on measures of cardiac structure and function. The data underlying this figure can be found in S12 and S13 Tables. LV, left ventricular; MR, mendelian randomization; MRI, magnetic resonance imaging. (PNG) [file pbio.3001656.s003.png]
