# Supplementary material for: A lifecourse mendelian randomization study highlights the long-term influence of childhood body size on later life heart structure
Source: PLoS Biol. 2022 Jun 9;20(6):e3001656. doi: 10.1371/journal.pbio.3001656 (PMC9182693; doi:10.1371/journal.pbio.3001656)
Supplement: Attachment — Submitted filename: Reviewer_responses.docx [file pbio.3001656.s020.docx]

**Reviewer #1:**

To the authors,

O'Nunain et al employed multivariable Mendelian randomization to investigate the potentially causal association of childhood body weight on adult cardiac size and function accounting for adult body weight. Their findings supported a direct effect of childhood body size on adult LV volumes and stroke volume in multivariable Mendelian randomization analyses accounting for adult body size. Similar results were observed accounting for fat-free mass index and birthweight, and findings direct associations with LV dimensions and wall thickness were observed in a replication analysis in the ALSPAC study. Parallel analysis using childhood and adult height demonstrated attenuated effect of height on LV measures after accounting for adult height, potentially pointing to childhood adiposity as particularly relevant. Parallel analysis for size of adult abdominal organs did not demonstrate evidence of an effect of childhood body weight as accounting for adult body weight.

The manuscript effectively uses multivariable Mendelian randomization analysis to help disentangle the impact of childhood vs adult body size on adult cardiac size. My comments are as follows:

1. The uploaded Supplementary Tables file only contains 2 unlabeled tables. Supplement tables 1 through 14 are not available to view to this reviewer. This a significant limitation to review since much supportive data is Supplementary.

**Many thanks for your comments on our manuscript. We apologise that the editors of Circulation did not provide the Supplementary Tables as an Excel file to you for this review. We attached the updated Supplementary Tables file after updating them in line with all comments in this review.**

2. As the investigators acknowledge, there are clear limitations in the assessment of childhood body size and height available in UKBB. However, it appears that the genetic variants used were validated with objectively measured BMI in ALSPAC, HUNT, and Finn study. This should be clarified in the Methods section.

**The validation of our genetic instruments with measured BMI in the 3 cohorts mentioned are described on page 6 in the Methods:**

‘Genetic instruments derived from these GWAS have been previously validated using measured BMI data from three independent populations; the Avon Longitudinal Study of Parents and Children (ALSPAC)^14^, the Trøndelag Health (HUNT) study^15^ and the Cardiovascular Risk in Young Finns Study^16^. Furthermore, genetic correlation analyses demonstrate that the childhood body size GWAS is much more highly correlated with measured childhood obesity from an independent sample (rG=0.85) compared to the adult measure (rG=0.67). In contrast, results from the adult body size GWAS have been shown to be much more strongly correlated with measured BMI in adulthood (rG=0.96) compared to the childhood measure (rG=0.64).’

3. The authors state that "the genetically predicted effect of childhood height on cardiac structure did not remain robust after accounting for height during adulthood" and that these findings suggest an effect of adiposity on cardiac structure, as opposed to just body size. However, while effect estimates for childhood height (and adult height) attenuate with multivariable MR models, they do remain significant suggesting some direct effect of childhood height on adult cardiac size. I think the argument for childhood adiposity as directly effecting adult heart size is perhaps less robust than the authors argue.

**As suggested by reviewer #2, we have now conducted further analyses to investigate the direct effect of childhood body size on cardiac structure accounting for the effect of childhood height using multivariable MR. The strong evidence of an effect of childhood body size on cardiac structure phenotypes was found with estimates only modestly attenuated. These results are described on page 14:**

‘Finally, evidence of an effect of childhood body size on measures of cardiac structure remained strong in the multivariable model accounting for the effect of childhood height (Supplementary Table 14).’

**Furthermore, we have emphasized in our conclusion that this finding requires confirmatory evidence from future research (page 18):**

‘Taken together, the evidence of a genetically predicted effect of childhood body size on cardiac structure found in this study may be driven by adiposity rather than these alternate aspects of body composition, although confirmatory evidence from further research is required to support this.’

4. The lack of evidence of direct effect of childhood body size on cardiomyopathy risk is interesting. Further discussion would be helpful as to how the authors interpret this finding. Does this suggest that adult body size is most relevant for cardiomyopathy risk and that whatever direct effects childhood body size have on adult cardiac size may not be inherently pathologic?

**We agree that additional interpretation of this finding would be useful for the reader. This has been added to page 16 of the discussion:**

‘These results suggest that the total effect of childhood body size is likely attributed to the long-term consequence of remaining overweight throughout the lifecourse and into adulthood. Likewise, although childhood body size increased the risk of cardiomyopathy there was no convincing evidence that it directly influenced risk of cardiomyopathy in later life (i.e. independently of adult body size).’

**And on page 17 of the discussion:**

‘We also build on these findings in this study, as evidence that childhood body size increases risk of non-ischaemic cardiomyopathy from our univariable analyses did not remain robust to the inclusion of adult body size in the multivariable model contradicting findings from observational analyses. These findings suggest that individuals who are larger in early life are likely at higher risk of non-ischaemic cardiomyopathy in later life due to a sustained and long-term effect of adiposity for many years across the lifecourse. However, this research question would be worthwhile revisiting once larger number of cases for cardiomyopathy endpoints are available^23^.’

5. A table describing prevalence of each childhood body size response, adult characteristics, and distribution of CMR variables would be helpful to contextualize the study.

**Thank you for this suggestion. We have now included a table of descriptive characteristics for the variables analysed in our study to Supplementary Table 2 (as referenced on page 8):**

‘Characteristics of these datasets can be found in Supplementary Table 2.’

**Reviewer #2:**

The manuscript by O'Nunain and colleagues describes a Mendelian randomization to look at the causal association of childhood body size and later life heart structure. Overall, the analyses are well-done, and the conclusions that childhood body size has a lifelong impact on heart structure, but not on other measures of adulthood organ size are fat percentage are well-supported. There are a few minor issues that should be addressed:

1) For the analysis of childhood body size on abdominal organ size, the authors statement the multivariable MR results for adult body size are robust except for pancreatic fat percentage and volume does not match the data. Volume was not significant in the univariable analysis to begin with, and fat percentage is still significant in the multi-variable MR.

**Thank you for this suggestion. We have now removed this statement in line with your comment.**

2) How do the authors explain the reversal in direction for liver fat and VAT volume in the multivariable estimates (Figure 2). Is this suggesting a causal association in the reverse direction, or some statistical artifact from the multivariable analysis?

**The reversal of direction in the multivariable MR model for childhood body size has been found when these scores have been applied to outcomes in separate investigations (for example with respect to heart failure and atrial fibrillation here** [**https://www.ahajournals.org/doi/10.1161/JAHA.121.021503**](https://www.ahajournals.org/doi/10.1161/JAHA.121.021503)**). These findings suggest that the total effect of childhood body size on liver fat and VAT as presented in the univariable MR model in this current study are predominantly attributed to young individuals remaining overweight throughout the lifecourse as described on page 15:**

‘Additionally, as anticipated evidence of a genetically predicted effect of childhood body size on adult measures of abdominal organ size and fat percentage attenuated after accounting for body size during adulthood. These results suggest that the total effect of childhood body size is likely attributed to the long-term consequence of remaining overweight throughout the lifecourse and into adulthood.’

**We believe however that claiming that there may be a ‘favourable effect’ of childhood body on these outcomes is too strong to make based on the evidence presented in our study. Likewise, conditional F-statistics for these instruments suggest that there is unlikely to be weak instrument bias in our multivariable MR analyses (page 6):**

‘Conditional F-statistics generated for childhood (F=13.6) and adult (F=16.0) body size instruments suggested that weak instrument bias was unlikely for these sets of genetic variants.’

3) Supplementary Figure 1 should have the order of the traits fixed so that both panels match.

**Thank you for this suggestion. We have now updated Supplementary Figure 1 accordingly.**

4) Not really convinced of the strength of the claim that childhood adiposity is key, and that height was not "robust" as a predictor. The childhood height associations were attenuated in the multi-variable MR, but still significant. Seems like if you want to claim adiposity is the driver, and that height plays a minimal/no role, you would need a multi-variable MR that include height and BMI (or some other adiposity measure).

**We have conducted the analysis you have suggested and strong evidence of an effect of childhood adiposity on cardiac structure was found. These results are now reported on page 14:**

‘Finally, evidence of an effect of childhood body size on measures of cardiac structure remained strong in the multivariable model accounting for the effect of childhood height (Supplementary Table 14).’

**Furthermore, we have emphasized in our conclusion that this finding requires confirmatory evidence from future research (page 18):**

‘Taken together, the evidence of a genetically predicted effect of childhood body size on cardiac structure found in this study may be driven by adiposity rather than these alternate aspects of body composition, although confirmatory evidence from further research is required to support this.’
