# Supplementary material for: A lifecourse mendelian randomization study highlights the long-term influence of childhood body size on later life heart structure
Source: PLoS Biol. 2022 Jun 9;20(6):e3001656. doi: 10.1371/journal.pbio.3001656 (PMC9182693; doi:10.1371/journal.pbio.3001656)
Supplement: Attachment — Submitted filename: Editorial_comments.docx [file pbio.3001656.s021.docx]

(A) After discussions within the editorial team, we would like to consider your manuscript as a Discovery Report. The editorial criteria for this article type can be found at the following link. (<https://journals.plos.org/plosbiology/s/what-we-publish#loc-discovery-report>).

During resubmission, we ask that you please change your submission to this article type.

**We have reformatted our manuscript as a Discovery Report as suggested.**

(B) We would to suggest the following modification to the title, to make it more compelling for our broad readership:

"A lifecourse Mendelian randomization study reveals that childhood body size has a long-term influence on later life heart structure"

**We have updated the title of this manuscript as suggested but with a slight tweak which we hope will be suitable - “A lifecourse Mendelian randomization study highlights the long-term influence of childhood body size on later life heart structure”**

(C) In your ethics statement, please include the approval number provided by the ethics committee that approved the study.

**We have now included approval numbers provided by ethics committees to the ethics statement:**

*‘Written informed consent was obtained for all study participants. Ethical approval for this study was obtained from the ALSPAC Ethics and Law Committee and the Local Research Ethics Committees and data were accessed under application C2823. Data from the UK Biobank (UKB) was accessed under application #15825 which has obtained ethics approval from the Research Ethics Committee (REC; approval number: 11/NW/0382).’*

(D) You may be aware of the PLOS Data Policy, which requires that all data be made available without restriction: <http://journals.plos.org/plosbiology/s/data-availability>. For more information, please also see this editorial: <http://dx.doi.org/10.1371/journal.pbio.1001797>

Note that we do not require all raw data. Rather, we ask that all individual quantitative observations that underlie the data summarized in the figures and results of your paper be made available in one of the following forms:

- Supplementary files (e.g., excel). Please ensure that all data files are uploaded as 'Supporting Information' and are invariably referred to (in the manuscript, figure legends, and the Description field when uploading your files) using the following format verbatim: S1 Data, S2 Data, etc. Multiple panels of a single or even several figures can be included as multiple sheets in one excel file that is saved using exactly the following convention: S1_Data.xlsx (using an underscore).

**We have reformatted our supplementary files so that they are all uploaded as individual files e.g. S1 Figure.png, S1 Table.xlsx. These files have been referenced individually in the manuscript e.g. S1 Table.**

- Deposition in a publicly available repository. Please also provide the accession code or a reviewer link so that we may view your data before publication.

Regardless of the method selected, please ensure that you provide the individual numerical values that underlie the summary data displayed in the following figures, as they are essential for readers to assess your analysis and to reproduce it.

Figure 2A-B

NOTE: the numerical data provided should include all replicates AND the way in which the plotted mean and errors were derived (it should not present only the mean/average values).

**We have now referenced the tables where the underlying data can be found for these figures:**

*‘The data underlying this figure can be found in Tables S3, S4, S6 and S7.’*

(E) Please also ensure that each of the relevant figure legends in your manuscript include information on *WHERE THE UNDERLYING DATA CAN BE FOUND*, and ensure your supplemental data file/s has a legend.

**As above we have now referenced the tables where the underlying data can be found for our Figure 2:**

*‘The data underlying this figure can be found in Tables S3, S4, S6 and S7.’*

**Legends have also now been added to add supplementary figures and tables.**

(F) Please ensure that your Data Statement in the submission system accurately describes where your data can be found and is in final format, as it will be published as written there. This includes referencing where the underlying data can be found in the Supplementary Information, as well as providing the accession numbers for any data deposited in public databases.

**We have updated our Data Statement on the submission site to describe where underlying data can be located in our Supplementary Information.**
